# Revisiting the Pathogenesis of Type 1 Diabetes: Importance of Neural Input to Pancreatic Islets and the Therapeutic Capability of Stem Cell Educator ^TM^ Therapy to Restore Their Integrity

**DOI:** 10.3390/biomedicines11020594

**Published:** 2023-02-16

**Authors:** Yong Zhao, Boris Veysman

**Affiliations:** Throne Biotechnologies, Paramus, NJ 07652, USA

**Keywords:** type 1 diabetes, autoimmune, islet innervation, parasympathetic nerve, sympathetic nerve, islet beta cells, immune modulation, Stem Cell Educator therapy

## Abstract

Type 1 diabetes (T1D) is an autoimmune disease with a shortage of islet β cells. To date, the etiology of T1D remains elusive. Increasing clinical evidence and animal studies demonstrate that autoimmune cells are directed against the nervous system of pancreatic islets, contributing to the development of T1D. Therefore, it highlights the necessity to explore novel clinical approaches to fundamentally correct the T1D autoimmunity not only focusing on islet β cells but also on protecting the islet nervous system. This allows the restoration of the integrity of islet innervation and the normal islet β-cell function. To address these issues, we developed a novel technology designated the Stem Cell Educator ^TM^ therapy, based on immune education by human cord-blood-derived multipotent stem cells (CB-SC). International amulticenter clinical trials demonstrated its clinical safety and efficacy to treat T1D and other autoimmune diseases. Stem Cell Educator ^TM^ therapy may have the potential to revolutionize the treatment of T1D, without the safety and ethical concerns associated with conventional immune and/or stem cell-based therapies.

## 1. Introduction

The human endocrine system consists of a group of fully-differentiated glands that are distributed in the body. Although the glands are small in size, they play essential roles in controlling cellular growth, differentiation, and development through their release of hormones. For instance, the pineal gland is a small, pinecone-shaped gland located in the center of the brain responsible for the highly conserved nighttime secretion of melatonin to regulate circadian rhythms [1]. Human pancreatic islets are scattered among the exocrine tissues of the pancreas with a diameter of about 100 μm/islet, comprising insulin-producing β cells, glucagon-producing α cells, pancreatic polypeptide-producing PP cells, somatostatin-producing δ cells, and ghrelin-producing ε cells [2,3,4,5]. To effectively regulate the body function at the holistic level, the endocrine system physiologically interacts with the nervous system and the immune system and forms an endocrine–neuro–immune (ENI) network through the endocrine/paracrine/autocrine signaling pathways, the distribution of central/peripheral nerve fibers, neuropeptides, cytokines, chemokines, and their receptors’ circuits [6,7]. Loss in the balance of the ENI network causes multiple autoimmune diseases [6,8,9].

Type 1 diabetes (T1D) is a serious autoimmune and metabolic disease caused by the autoimmune destruction of pancreatic islets leading to the deficit of islet β cells [10,11]. Millions of individuals worldwide have T1D, and its incidence has markedly increased since the COVID-19 pandemic [12,13]. Although daily insulin injections offer limited control over blood sugar levels and may delay the onset of chronic complications due to dysglycemia, a true cure has proven elusive despite intensive research efforts over the past 40 years. Recent clinical trials have highlighted the limits of conventional immunotherapy and underscore the need for novel approaches to fundamentally address autoimmunity through advanced understanding of the pathogenesis of T1D. T1D etiology appears to be multifactorial, including genetic, epigenetic, physical, social, and environmental factors, leading to the dysfunctions of multiple immune cell compartments such as T cells, B cells, regulatory T cells (Tregs), monocytes/macrophages (Mo/Mϕ), dendritic cells (DC), natural killer (NK) cells, and natural killer T (NKT) cells [14,15]. Mechanistic studies in non-obese diabetic (NOD) mice confirmed that T-cell-mediated autoimmune destruction of islet β cells is initiated by type 1 F4/80^+^CD11c^+^ islet macrophages [16,17,18]. However, accumulated evidence challenged this traditional concept of T cell/macrophage-mediated pathogenesis in T1D autoimmunity [19,20,21,22]. As a part of the ENI network of pancreatic islets, islet-resident macrophages (Mϕ) not only function as antigen-presenting cells (APC) responsible for an innate immunity, but also play an important role in the development of pancreatic islets [16]. The op/op mice lacking functional macrophage colony-stimulating factor (M-CSF, also termed CSF1) show the reduced islet size [16] in addition to the reduction in macrophage numbers per islet [16]. Additional clinical evidence and animal studies demonstrated that there is damage or loss of the pancreatic islet nervous system in newly-diagnosed T1D patients [23] and in autoimmune-caused diabetic NOD mice and Bio-Breeder rats [24,25,26]. Therefore, the fine tuning of the balance of this local ENI network is necessary to maintain normal islet function and metabolic control. Here, we summarize the current progress in understanding the pathogenesis of T1D in order to find a cure for T1D. 

## 2. Pancreatic Islet Innervation Contributes to the Normalization of Islet β-Cell Function

Human pancreatic islets are primarily regulated by two autonomic nervous systems including the parasympathetic and sympathetic nervous systems [27,28]. The parasympathetic nervous system (vagus nerve) is derived from the central nervous system and penetrates into islets, promoting the insulin secretion from β cells and reduces hyperglycemia [27,29,30,31]. In contrast, the sympathetic nervous system originates from the paravertebral sympathetic ganglion chain and stimulates the glucagon secretion from α cells, leading to the increased level of blood glucose [27]. Both the vagus nerve and the sympathetic nerve can be further characterized into afferent and efferent nerve fibers, respectively [32]. There are five types of parasympathetic neurotransmitter muscarinic acetylcholine receptors (mAChRs), which are characterized as M1–M5 and expressed on different types of neurons and tissue cells in humans [33]. Importantly, distinct islet cells display different types of mAChRs. There are high levels of M3 and M5 expressions on the islet β cells in the pancreata of the Caucasian population [34]; islet α cells display the M2 receptor and produce a cholinergic signal that contributes to the modulation of islet β-cell function via the paracrine pathway [35]. Interestingly, our previous studies revealed the molecular disparities of expression of mAChRs on human islet β cells between Caucasian and Chinese populations [36]. The M2 receptor and other specific cholinergic markers such as vesicular acetylcholine transporter (vAChT) and choline acetyltransferase (ChAT) are markedly expressed on the islet β cells of the pancreata of Chinese populations [36]. 

The role of the nervous system in the functioning of pancreatic islets has been recognized for decades. Rodriguez-Diaz et al. reported autonomic axons (e.g., parasympathetic and sympathetic) in human islets with unique innervation patterns, with the invading sympathetic fibers preferentially innervating smooth muscle cells of the blood vessels located within the islet [37]. There were high concentrations of γ-aminobutyric acid (GABA, a prominent inhibitory neurotransmitter in central nervous system) distributed in the cytoplasm of islet β cells, with a small percentage of β-cell GABA located inside the insulin granules [38]. Sorensen et al. found the presence of GABAergic nerve cell bodies at the periphery of pancreatic islets with numerous GABA-containing processes extending into the islet mantle [38]. Saravia-Fernandez et al. observed the same structures in NOD mice and showed that these structures also express neuropeptide Y (NPY) [39]. NYP innervation of pancreata has been shown previously in a variety of species. Such neuronal networks are critical for pancreatic islets to maintain their homeostasis and metabolic controls. 

## 3. Cross-Reaction of T1D Autoimmunity between Islet β-Cells and Autonomic Nerves

T1D has been widely thought of as the T cell-mediated autoimmune destruction of islet β cells. Until recently, Leete et al. found that different age groups of T1D subjects exhibited different mechanisms of pathogenesis [20,21]. All subjects within the younger age group (<7 years old) uniformly displayed a high profile of CD20^+^ B cells (CD20Hi) in the inflamed islets in addition to the preponderance of CD8^+^ T cells [20]. Due to the aggressive nature of these immune cells, this group tends to lose their islet β cells at a rapid rate. However, those who received a diagnosis at an older age (≥13 years of age) had a low profile of CD20^+^ B cells (CD20Lo) and therefore a less aggressive disease progression. Patients in whom T1D developed between the age of 7 and 12 years fell into either group [20]. This indicates that there were different profiles of immune cells contributing to the insulitis of T1D in different age groups. To characterize the antigenic repertoire of islet-infiltrating B-cells in T1D, Carrillo et al. generated hybridoma cell lines of islet-infiltrating B-cells from nonobese diabetic (NOD) mice and NOD mice expressing a diabetogenic T-cell receptor (8.3-NOD) [24]. Surprisingly, characterization of the tissue antigenic specificity of islet-infiltrating B-cells in pre-diabetes is predominantly directed against the nervous system elements of the pancreatic islets [24]. 

Although islet β cells have been selectively destroyed by autoimmune cells in T1D, many target autoantigens are not exclusively expressed by β cells but are shared with the nervous or neuroendocrine systems. Such autoantigens are glutamic acid decarboxylase (GAD)-65 [40], protein tyrosine phosphatase such as protein IA-2 [41], and zinc transporter 8 (ZnT8) [41,42]. Winer et al. reported the spontaneous autoimmune targeting of pancreatic nervous system tissue elements, peri-islet Schwann cells (pSC), in both diabetes-prone humans and early in prediabetic NOD mice [43]. Similar autoantibodies and T-cell autoreactivities to the pSC-expressed antigens such as glial fibrillary acidic protein (GFAP) and S100β were found in humans with probable prediabetes and young NOD females [43], indicating that autoimmune targeting pSC may represent an earlier pathogenic process for the initiation of T1D. In line with this, Mundinger et al. demonstrated that T1D patients displayed an early, marked, sustained, and islet-selective loss of sympathetic nerves [23]. Both very recent onset (<2 weeks) and long duration (>10 years) T1D patients have a severe loss of islet sympathetic nerves [23]. In contrast, type 2 diabetic patients failed to show the loss of islet sympathetic nerves [23]. Using vesicular monoamine transporter 2 (VMAT2) as a marker for sympathetic nerve terminals, Mei et al. showed a marked decrease in the VMAT2-positive nerve fiber area in the islets of new onset diabetic BB rats relative to their nondiabetic controls [25]. However, there was no decrease in the fiber area of sympathetic nerves in the chemical streptozotocin (STZ)-induced diabetic rats relative to that of the control group [25]. Interestingly, using the 3D panoramic histology, Tang et al. reported the increased density of pancreatic sympathetic nerves in the hyperphagic db/db mice at weaning age [44]. These data suggest that the loss of sympathetic nerves was associated with the autoimmunity. In agreement with this view, Taborsky et al. demonstrated the loss of sympathetic nerves in pancreatic islets, which was coupled with the invasive insulitis in autoimmune-caused diabetic NOD mice [26]. The loss of islet sympathetic nerves progressed with the duration of diabetes. Importantly, the neuropeptide Y (NYP)-positive nerve fiber area in the islets of cyclophosphamide-induced diabetic NOD mice was markedly less than that in the age- and sex-matched non-diabetic NOD mice, and that of naturally-onset diabetic NOD mice [26], because cyclophosphamide-induced diabetes in the NOD mice was associated with a reduction in CD4^+^CD25^+^Foxp3^+^ regulatory T cells (Tregs) and accelerated the autoimmune destructions [45]. 

## 4. Infiltration of Autoimmune Cells against the Islet Nerves

**Figure 1:**
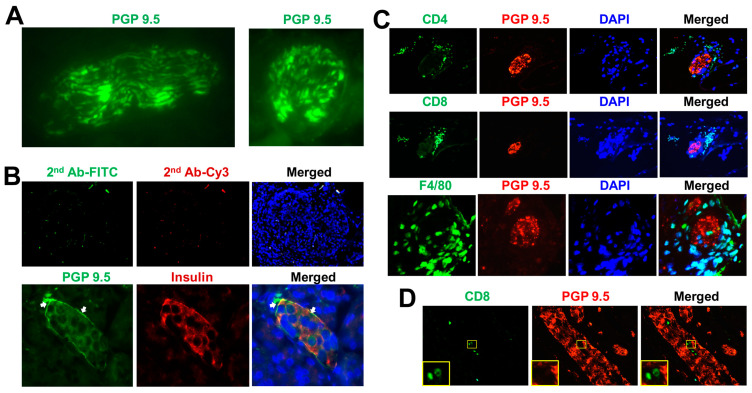
Infiltration of autoimmune cells to pancreatic nervous system. (**A**) The bundles of PGP 9.5-positive nerve fibers are shown by the immunohistochemistry of human pancreata with the vertical section (left) and horizontal section (right). (**B**) Innervation of human pancreatic islet with the distribution of PGP 9.5-positive (green) neuronal fiber around insulin-positive islet β cells (red). The 2nd Ab immunostaining served as the negative control. Original magnification, ×600. (**C**) Infiltration of immune cells such as CD4^+^ T cells, CD8^+^ T cells, and F4/80^+^ macrophages (green) to the PGP 9.5-positive nerve fibers (red) in pancreata of humanized diabetic NOD-scid IL-2rγ^null^ mice. (**D**) The CD8^+^ T cells directly targeted the PGP 9.5-positive nerve fiber (red), with a high magnification shown in the insert. The irradiated spleen mononuclear cells (SMC) of diabetic NOD mice were adoptively transferred into NOD-scid IL-2rγ^null^ mice (1 × 10^7^ cells/mouse in 300 μL PBS, i.p.). Original magnification, ×600.

The above studies in diabetic animals and patients indicated that autoimmunity played a critical role in the loss of islet nerve innervation and the development of T1D. To provide evidence that autoimmune cells directly attack the nerve fibers of pancreatic islets, we developed a humanized immune cell-mediated T1D model in NOD-scid IL2rγ^null^ mice [46]. The selective destruction of islet β cells was achieved by human T cells after an initial trigger provided by the injection of irradiated spleen mononuclear cells from diabetic NOD mice. This resulted in severe insulitis, a marked loss of total islet β-cell mass and other diabetic-related phenotypes [46]. Using a neuroendocrine cell marker protein gene product 9.5 (PGP 9.5) [47], further immunohistochemistry analysis demonstrated the direct infiltration of autoimmune cells from diabetic NOD mice to the PGP 9.5-positive pancreatic nerve fibers, which included F4/80^+^ macrophages, CD4^+^ and CD8^+^ T cells (Figure 1), and might cut off the “wire” (nerve fiber) to pancreatic islets by these infiltrated immune cells. Prospectively, the neuropathy of islet innervation could be directly caused by these infiltrated immune cells and/or their released inflammatory cytokines, leading to the development of diabetes in NOD-scid IL-2rγ^null^ mice [46]. Alternatively, due to the shortage of neurotrophic support, the loss of the integrity between the islet nerve system and β cells may induce and result in the apoptosis or necrosis of islet β cells, which in turn activate the islet macrophages to evoke the infiltration of dendritic cells (DC), T and B cells, and damage in pancreatic islets. 

## 5. Reversal of T1D by the Treatment with Stem Cell Educator ^TM^ Therapy

### 5.1. Correct the Autoimmunity through the Induction of Immune Tolerance by Stem Cell Educator ^TM^ Therapy

**Figure 2:**
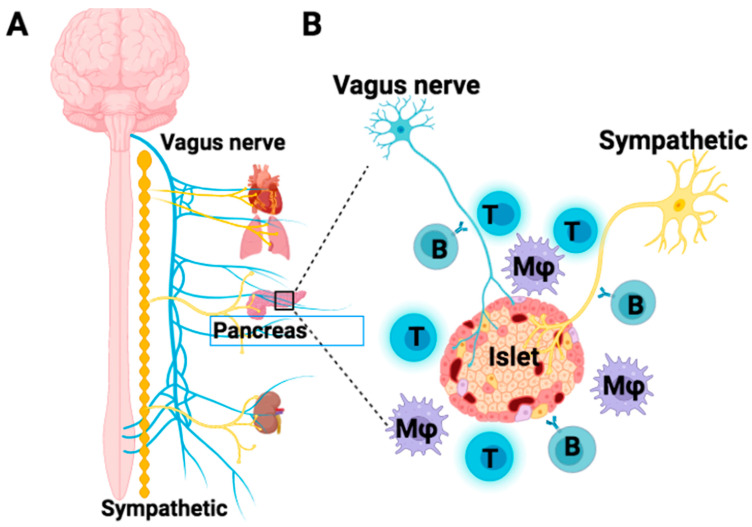
Scheme of the infiltration of autoimmune cells against pancreatic islets and autonomic nerves. (**A**) Innervation of vagus nerve fibers (blue) and sympathetic nerve fibers (yellow) from the paravertebral sympathetic ganglion chain (orange) into major organs including heart, lung, kidney, and pancreas (left panel). (**B**) Different types of immune cells attack the islet, vagus nerve (blue), and sympathetic nerve (yellow).

Animal and clinical studies have demonstrated the loss of islet innervation caused by the infiltrated autoimmune cells (Figure 2). Therefore, it is necessary to protect islet innervation and restore the integrity between pancreatic islets and the nerve system to fundamentally correct the autoimmunity for T1D treatment. Due to the potential of axon growth [48], it is expected that the islet nerve fibers may be elongated and branched around islets after the autoimmunity is controlled, and this re-innervation may lead to the replication of residual islet β cells and restoration of β-cell function. In line with this expectation, our previous animal studies demonstrated that the treatment of established autoimmune-caused diabetes in non-obese diabetic (NOD) mice with the purified autologous CD4^+^CD62L^+^ Tregs co-cultured with human cord blood-derived multipotent stem cells (CB-SC) could reduce hyperglycemia, promote islet β-cell proliferation to increase β-cell mass and insulin production, and reconstitute islet architecture [49]. 

To find a cure for patients with T1D and other autoimmune diseases, we developed the Stem Cell Educator ^TM^ therapy by using a new type of cord blood-derived multipotent stem cells (CB-SC). With this innovative technology, a patient’s blood is circulated through a blood cell separator, where the patient’s immune cells are co-cultured with adherent CB-SC in vitro and “educated” immune cells are returned to the patient’s circulation. Notably, following the Stem Cell Educator ^TM^ therapy, long-standing established T1D patients increased their C-peptide levels (a by-product of insulin production) of both fasting and post glucose challenging [11]. According to the Juvenile Diabetes Cure Alliance (JDCA, New York), Stem Cell Educator ^TM^ therapy is ranked the leading practical cure project among 607 global type 1 diabetes projects in 2022. Comparing with other technologies (Table 1), Stem Cell Educator ^TM^ therapy displays several advantages to fundamentally correct the autoimmunity and restore the immune tolerance through the expression of key transcription factor autoimmune regulator (AIRE) in CB-SC and other molecular/cellular mechanisms, without rejection issues [10]. Over the last 12 years, international multicenter clinical trials in the United States, China, and Spain have strongly demonstrated the clinical safety and efficacy of Stem Cell Educator ^TM^ therapy in more than 200 patients aged from 3 to 75 years old, which have demonstrated its long-lasting clinical safety and efficacy in the restoration of islet β-cell function in T1D patients [11,36,50,51] and the treatment of other autoimmune diseases (e.g., alopecia areata [52], psoriasis, and lupus).

Substantial evidence demonstrates that autoimmune memory T cells constitute the most significant barriers to curing autoimmune diseases, including T1D. Our ongoing clinical trials and previous studies demonstrated the therapeutic capability of Stem Cell Educator ^TM^ therapy to correct the autoimmune memory in T1D [36] and other autoimmune diseases [10]. Delgado et al. showed that the percentages of CD4^+^ T_EM_ and CD8^+^ T_EM_ cells were substantially decreased in the peripheral blood of T1D subjects who had received Stem Cell Educator^TM^ therapy; CD4^+^ T_CM_ cells were not impacted [36]. Furthermore, Stem Cell Educator ^TM^ therapy led to increased levels of CCR7 expression on naïve T and T_CM_ cells and increased percentages of CCR7^+^ T_CM_ at the expense of CCR7^−^ T_EM_ [36]. Together, these findings show that Stem Cell Educator ^TM^ therapy targets T cells for modulation, rather than destruction, restoring populations of naïve T cells and eliminating those responsible for autoimmune responses.

To date, our preclinical [49,50,53,54,55] and clinical [11,36,50,51,52] studies have identified the following cellular and molecular players mediating the immuno-modulating activities of CB-SC during Stem Cell Educator ^TM^ therapy [10]: (1) expression of autoimmune regulator (AIRE) in CB-SC [11,56]; (2) cell–cell contacts mediated by PD-L1(CD274) [54] and HVEM (CD270), which are expressed on CB-SC [52] and whose ligands PD-1 and BTLA are expressed on immune cells (T cells, B cells, monocytes, DC, and granulocytes) [52]; (3) soluble factors (nitric oxide, TGF-β1) released by CB-SC [54]; (4) modulating interactions between antigen-presenting cells (e.g., Mo/Mϕ) and T cells through co-stimulatory molecules and their ligands [51]; (5) release of CB-SC-derived exosomes that polarize the type 2 macrophage (M2) differentiation of Mo/Mϕ [53,57]; (6) CB-SC displayed multiple immune modulations on B cells through the Galectin-9-mediated cell–cell contact mechanism [58]. Like the nerve fiber infiltration with the multiple types of immune cells, T1D-associated immune dysfunctions have been traced to different cell compartments, including T cells, B cells, regulatory T cells (Tregs), monocytes/macrophages (Mo/Mϕ), and dendritic cells (DC). Therefore, Stem Cell Educator ^TM^ therapy displays immune education on multiple immune cells, leading to the restoration of immune balance. 

Recently, Richardson et al. reported that there were pancreatic capillaries and nerve fibers persisting in both recent onset and longstanding T1D patients [59]. Additional studies showed that the number and densities of sympathetic nerves were decreased in the non-diabetic islet autoantibody-positive individuals [60] and T1D patients [23], without significantly affecting the islet-associate parasympathetic axons [61]. Therefore, it is necessary to protect and restore the islet innervation for the prevention and treatment of T1D. Importantly, by the collection of a patient’s own immune cells through apheresis that are “educated” by CB-SC [10], it is expected that Stem Cell Educator ^TM^ therapy may protect both the islet nervous systems and islets against the autoimmunity, leading to the restoration of islet ENI integrity and islet β-cell function for the treatment of T1D. Our previous studies have demonstrated this capability of improving islet β-cell function and proliferation in the autoimmune-caused NOD mice [49] and T1D patients after the treatment with CB-SC’s immune modulation [11,56]. 

### 5.2. Overcome the Shortage of Islet β Cells through the Alternative Approaches

It is essential to rescue the residual β cells for recent onset of T1D patients after correcting the autoimmunity with Stem Cell Educator ^TM^ therapy, which has been demonstrated by previous clinical trials [11,36]. Due to human islet β-cell replication usually occurring during the fetal and neonatal stages and then declining after these stages, it will be necessary to provide alternative resources for the restoration of β-cell function in those longstanding severe T1D subjects. Recently, functional insulin-producing cells have been generated from embryonic stem cells (ESC) and induced pluripotent stem cells (iPSC) [62,63,64]. This has led to clinical trials for the treatment of T1D subjects including ViaCyte studies with VC-01 and VC-02 products (NCT04678557 and NCT03163511, respectively) and a Vertex study with VX-880 (NCT04786262). Clinical applications of these stem-cell-derived insulin-producing cells may have ethical and safety concerns including potential tumor formation and immune rejection [65,66]. To circumvent the immune rejection, encapsulations of ESC- or iPSC-derived insulin-producing cells or using an immunosuppressive regimen have been tested in animal studies and clinical trials [67,68,69]. Recently, ViaCyte is trying to develop the gene-edited stem cell-derived therapy for the treatment of T1D in collaboration with CRISPR Therapeutics (NASDAQ: CRSP), a biotech company focusing on the development of transformative gene-based medicines for serious diseases. 

Using a similar approach previously utilized for an isolation of CB-SC [70], Zhao et al. characterized adult peripheral blood insulin-producing cells (PB-IPC) [71] from adult human peripheral blood, displaying islet β-cell-related markers (e.g., the expression of β cell-specific insulin gene transcription factors and prohormone convertases) and reducing hyperglycemia with migration to pancreatic islets after transplant into the chemical streptozotocin (STZ)-induced diabetic mice [71]. To improve the differentiation of PB-IPC toward islet β cells, PB-IPC were treated with the purified platelet-derived mitochondria [72,73]. Notably, Yu et al. generated the pluripotent stem cells from adult human peripheral blood PB-IPC following the treatment with platelet-derived mitochondria. Ex vivo and in vivo functional studies established that treatment with platelet-derived mitochondria can reprogram the transformation of adult PB-IPC into functional CD34^+^ hematopoietic (HSC)-like stem cells, leading to the production of blood cells such as T cells, B cells, monocytes/macrophages, granulocytes, red blood cells, and megakaryocytes (MK)/platelets [73]. Additionally, these mitochondrion-induced PB-IPC (miPB-IPC) can give rise to retinal pigment epithelium (RPE) cells and neuronal cells in the presence of different inducers [72], highlighting the pluripotent capability of the differentiation of PB-IPC into three-germ layer-derived cells. Through confocal and electron microscopy, Yu et al. found that exogenous mitochondria enter cells and directly penetrate the nucleus of PB-IPC where they can produce profound phenotypic changes [72]. Thus, these findings reveal a novel function of mitochondria directly contributing to cellular reprogramming, thus overcoming the limitations and safety concerns of using conventional technologies to reprogram embryonic stem (ES) and induced pluripotent stem (iPS) cells in regenerative medicine. In comparison with the generation of insulin-producing cells from ES and iPS cells, this technology can efficiently isolate insulin-producing PB-IPC cells from patients’ own blood, without any ethical issues or the hazards of immune rejection. Mitochondrial reprogramming of PB-IPC may provide a novel approach for the generation of a large amount of autologous insulin-producing cells from patients themselves to potentially treat T1D patients in clinics after further optimizing ex vivo differentiation protocol.

Our previous studies demonstrated PB-IPC naturally circulate in human peripheral blood [71,72,73]. The preliminary clinical data from our ongoing clinical trial (IND 19247) revealed that the percentage of PB-IPC was very low in T1D patients. Notably, its percentage was markedly increased after receiving Stem Cell Educator ^TM^ therapy. Therefore, the recovered PB-IPC may display therapeutic capability to replenish the damaged neuronal cells/nerve fibers and β cells due to their multiple differentiation potential. They could migrate into pancreatic islets via their expression of the chemokine receptor CXCR4/SDF-1 (stromal cell-derived factor-1) mechanism [71]. 

## 6. Conclusions

Human islets are well-vascularized and innervated organs with specialized cells to precisely release different hormones and maintain the homeostasis through the ENI networks at the systematic level and local networks of intrapancreatic islets. To date, clinical evidence and animal studies substantiate the functional importance of neural input to pancreatic islets. Both sympathetic and parasympathetic innervation of human islets contribute to the metabolic control and the regulation of hormone release. During the pathogenesis of T1D, this integrity and neural pathways of islets are damaged by the infiltration of autoimmune cells, leading to the collapse of islet microarchitectures (e.g., distribution and number of islet cells, vasculature, nerve fibers, neuronal projections, and extracellular matrix). Therefore, T1D pathophysiology is not only the selective destruction of the islet β cells but also the product of targeting of the islet nerve system by autoimmune cells. This concept needs to be updated to ultimately heal the T1D patients. To eliminate these autoimmune cells, it is essential to fundamentally overcome the autoimmune memory. Expanded clinical studies are required to further explore this capability through the ongoing FDA-approved clinical trial of Stem Cell Educator ^TM^ therapy in T1D (IND 19247, ClinicalTrials.gov Identifier: NCT04011020) and other potential approaches to comprehensively restore the integrity and neural pathways of functional islets. 

## Figures and Tables

**Table 1:** Compare the Stem Cell Educator ^TM^ therapy with other ongoing clinical therapies/trials in T1D.

List of Products	Company/ Hospital	ClinicalTrials.gov	Target the Autoimmunity		Restoration of β-Cell Function		
			Immune Modulation	Immune Suppression	Improve the Endogenous β-Cell Regeneration	Transplant Exogenous β-Cell Surrogates	Rejection and Need Immune Suppression
Stem Cell Educator ^TM^ therapy (Gleukocell ^TM^)	Throne	NCT04011020 Phase 2/3	Yes	No	Yes	No	No
VX-880 (ES cell-derived insulin-producing cells)	Vertex	NCT04786262 Phase 1/2	No	N/A	No	Yes	Yes
VC02-101 (ES cell-derived β-cell progenitors)	ViaCyte	NCT03163511 Phase 1/2	No	N/A	No	Yes	Yes
PIpepTolDC (vaccine therapy)	City of Hope	NCT04590872 Phase 1	Yes	N/A	N/A	No	No
TZIELD (teplizumab-mzwv)	Prevention Bio	FDA approved	No	Yes	N/A	No	No

## Data Availability

The data supporting the conclusions of this article are available from the corresponding author upon reasonable request. They are not publicly available due to ethical restrictions.
